# Effect of pre-consultation testing on clinicians’ adherence to malaria test results and waiting time among children under 5 years in the Northern Zone of Volta Region of Ghana

**DOI:** 10.1186/s12936-020-03189-6

**Published:** 2020-03-20

**Authors:** Jonathan Mawutor Gmanyami, Asiwome Ameko, Saviour Selase Ahiafe, Samuel Adolf Bosoka, Margaret Kweku, Evelyn Korkor Ansah

**Affiliations:** 1 School of Public Health, University of Health and Allied Sciences, Ho, Volta Region Ghana; 2 Centre for Malaria Research, Institute for Health Research, University of Health and Allied Sciences, Ho, Volta Region Ghana

**Keywords:** Pre-consultation testing, Post-consultation testing, Clinician, Adherence, Waiting time, Ghana, Malaria

## Abstract

**Background:**

The Ministry of Health, Ghana, in accordance with global policy, recommends that all suspected malaria cases be confirmed parasitologically before treatment. Not all clinicians, however, base their treatment on test results. Patients also spend a lot of time at health facilities waiting to consult a clinician before being asked to go for testing and to see a clinician with test results. The purpose of the study was to determine if testing all children aged 6 to 59 months with fever reporting at an outpatients department (OPD) for malaria before consultation with a clinician (pre-consultation testing) will influence clinicians to adhere to test results and also reduce the time spent by such patients.

**Methods:**

A quasi-experimental study design was used involving two randomly selected government-owned hospitals in the Northern Volta, Ghana. In each hospital, 439 children were recruited between November 2018 and January 2019. The intervention hospital implemented pre-consultation testing. In the comparator arm, standard practices, which involved patients seeing the clinician before he/she decides whether to send the patient for testing or not, were maintained.

**Results:**

Out of 878 children screened the overall prevalence of malaria was 31.9% by malaria rapid diagnostic test (RDT) and 26.7% by microscopy. Clinicians in the intervention arm adhered more to the malaria test results than those in the comparator arm (93.2 vs. 84.3%; p < 0.001). The proportion of children who tested negative but were still diagnosed with malaria was significantly lower in the intervention arm compared to the comparator arm (8.4 vs. 21.2%: p < 0.001). Clinicians and mothers/caregivers in both arms preferred pre-consulting testing. Six out of every 10 mothers/caregivers in the comparator arm viewed the waiting time as ‘too long’’ compared to 4 out of every 10 mothers in the intervention arm. On average, patient waiting time was significantly lower in the intervention arm (2.61 h) than in the comparator arm (3.42 h).

**Conclusion:**

Pre-consultation testing significantly improves clinicians’ adherence to malaria test results, shortens patients’ waiting time and leads to overall patient satisfaction. There is a need to establish RDT corners at OPDs of health facilities to implement pre-consultation testing.

## Background

Malaria is a deadly disease caused by protozoan parasites of the genus *Plasmodium* that are transmitted to people through the bites of infected female *Anopheles* mosquitoes. There are five parasite species that cause malaria in humans, however, *Plasmodium falciparum* and *Plasmodium vivax* pose the greatest threat, with *Plasmodium falciparum* being the most prevalent and the cause of most malaria-related deaths on the African continent [1]. In Ghana, the main parasite species that cause malaria are *P. falciparum* (80–90%), *Plasmodium malariae* (20–36%) and *Plasmodium ovale* (0.15%) [2].

According to Ezeonwu et al. [3] preventable infections, including malaria, are the major causes of morbidity and mortality in children under 5 years old. In regions with high transmission of malaria, children under 5 years of age are most susceptible to infection and death with more than two-thirds (70%) of all malaria deaths occurring in this age group [4].

Malaria is endemic in all regions of Ghana with a reported prevalence of 28% among children aged 6–59 months, based on microscopy [5]. The Volta Region, one of the 16 administrative regions of Ghana, reported the third highest (28%) malaria prevalence in Ghana [5]. Strategies to control and eliminate malaria morbidity, mortality and transmission include rapid and accurate diagnosis, as well as prompt and adequate treatment [6]. It is the most effective intervention to prevent a mild case of malaria from developing into severe disease and probable death. The World Health Organization (WHO) recommends prompt parasitological confirmation either by microscopy or rapid diagnostic test (RDT) for all patients with suspected malaria [4], and treatment of confirmed cases of uncomplicated falciparum malaria with artemisinin-based combination therapy (ACT) [1]. However, the diagnosis of malaria using microscopy is time-consuming, labour-intensive and expensive [7]. There is a lack of reliable and suitable microscopy in most peripheral health centres [8] and prompt parasitological confirmation of malaria before treatment is unrealistic in many settings because expert laboratory diagnostic services are scarce or unavailable [9]. On the other hand, clinical diagnosis based on signs and symptoms of malaria has proven to be non-specific and leads to an increased indiscriminate use of anti-malarial drugs due to over-treatment of malaria or non-treatment of other diseases in malaria-endemic areas [9–11]. These shortcomings of clinical diagnosis and microscopy have favoured the implementation and use of RDTs, which allow malaria diagnosis even in health facilities that lack any laboratory facility. RDT therefore serves as an important tool to implement WHO-recommended, parasite-based diagnosis in regions where expert microscopy is not available [12]. The role of RDT is important to reduce turnaround times (TAT) in situations where there is a very busy facility [7].

In Ghana, malaria diagnosis is progressively being shifted from clinical to parasitological confirmation as the basis of treatment, and this is in compliance with global initiatives and recommendations such as the test, treat and track (T3) to scale up parasite-based diagnosis in all age groups [2]. The initiative which includes testing all suspected cases, treating these cases with ACT, and tracking (monitoring) malaria, reaffirms the importance of RDTs in malaria control. In 2014, the Ministry of Health (MOH) of Ghana indicated that every suspected case of malaria must be tested by RDT or microscopy prior to treatment as part of the guidelines for case management of malaria. RDTs are central to efforts towards decreasing malaria over diagnosis, the consequent overuse of valuable anti-malarial drugs and under-diagnosis of alternative causes of fever [13]. The practice of testing for malaria in Ghana is usually carried out after consulting with the clinician (post-consultation testing) which helps to reduce presumptive treatment; the challenge is that it tends to increase the time patients spend at a health facility, as they first wait in queue at an outpatients department (OPD), consult with the clinician, proceed to the laboratory for testing and finally return with the test results to the clinician for treatment [14]. In spite of this, RDTs still reduce the time spent at the OPD compared to microscopy and also results in a significant reduction in over-prescription of anti-malarial drugs [15].

Testing of patients immediately after triaging so that the patient sees the clinician with test results (pre-consultation testing) is likely to promote clinicians’ adherence to test results and also potentially shorten the waiting time at the health facility. The study therefore sought to assess whether implementation of pre-consultation testing would influence clinicians to adhere more to malaria test results, consider alternative diagnoses in the event of negative test results and lead to a reduction in time spent at the health facility by mothers/caregivers and their children age under 5 years in the Northern Zone of Volta Region, Ghana.

## Methods

### Study site and population

The study was carried out at two government-owned district hospitals in two districts that were randomly selected in the Northern Zone of Volta Region of Ghana from November 2018 to January 2019. The Volta region stretches across all the ecological zones of the country: Coastal Savannah, Middle forest and Northern/Sahel Savannah. The regional prevalence of malaria among children under five years of age decreased from 25.2% (2014) to 24.5% (2018) [5, 9]. The case fatality rate for malaria among children under 5 years of age in the region has also consistently decreased over the period 2016 to 2018, from 0.39 to 0.17 and to 0.13%, respectively.

The region has 326 health facilities, of which 242 are public sector owned, 18 mission owned, 1 quasi-government (military hospital), and 65 privately owned [16, 17]. The study involved two government-owned district hospitals in the northern zone of the region: Hohoe Government Hospital and Jasikan Government Hospital. These hospitals serve clients from other districts as well as neighbouring Togo and are the major referral centres along the stretch of the eastern corridor.

The government hospitals in the Northern Zone of Volta Region were paired according to certain characteristics, including size, caseload, staffing, and availability of testing facilities. The names of each pair of hospitals were written on pieces of paper, wrapped into small balls and placed in a container. An independent person not participating in the study was asked to pick one wrapped paper from the container. The names of the randomly selected hospital pair were subsequently written on two separate pieces of paper and an independent person again asked to pick one. The hospital that was picked became the intervention hospital while its counterpart served as the comparator hospital.

The study population comprised children aged 6 to 59 months with complaint of fever or history of fever reporting to the two government-owned hospitals as well as clinicians providing health care in those hospitals.

### Study procedures

A quasi-experimental study design was used for the study, which involved two randomly selected government-owned district hospitals, one of which was the setting for the intervention arm and the other, the setting for the comparator arm from November 2018 to January 2019. Trained nurses and data collectors assisted in data collection. The informed consent forms were translated into two local languages and administered to participants based on the language they were most comfortable with.

For the intervention arm, an RDT corner was set up at an OPD to test all children aged 6 to 59 months complaining of fever or a history of fever before consultation with the clinician so that the clinician could see the patient with their test results. In the comparator arm, standard services that involve patients seeing a clinician before he/she decides whether to send the patient for testing or not, were maintained. In the laboratory in that setting when blood samples were being taken for microscopy, RDT was done concurrently. In the comparator arm, if after consultation with the patient, the clinician does not request for malaria testing but diagnoses an eligible child based on clinical symptoms, such child was excluded from the study.

Data collection methods employed were quantitative. Questionnaires were administered to caregivers of children and clinicians providing care to the children under 5 years old. Clinicians were interviewed after the estimated sample size of mothers/caregivers was recruited, to avoid them being aware that the test results presented during the consultation was to determine their level of adherence to the test results. This was done to avoid bias.

The dependent variables were clinicians’ adherence to test results and time spent in the health facility by the children. The independent variables considered in this study comprised socio-demographic factors (such as gender and age of child, ownership and use of long-lasting insecticidal nets (LLIN) among the children, age of parent/guardian of child, geographic location, education level, and occupation) and other epidemiologic factors (fever or history of fever (within 48 h).

Based on the prevalence of malaria among children under 5 years of age by RDT in the Volta region [5], a precision of 5%, and a confidence interval of 95%, the estimated sample size was a total of 878 with 439 children to be recruited from each hospital. The sample size was calculated with the intention to show the difference in the adherence to malaria test results between the two study arms. The clinicians who attended to the children were purposively selected.

### Diagnosis of malaria by RDT

Trained nurses and laboratory technicians in the study arms tested children aged 6 to 59 months for malaria using the RDTs. In the intervention arm, trained nurses tested for malaria using RDT before consultation with the clinician, while in the comparator arm, the children were tested in the laboratory after consultation with the clinician.

The SD BIOLINE Malaria Ag P.F (HRP2/pLDH) test kits were used for the rapid qualitative detection of histidine-rich protein II (HRP-II) antigen and lactose dehydrogenase (pLDH) from malaria *P. falciparum* in the finger prick blood sample in the diagnosis of malaria infection. Each child was given a specific identification number (ID). The unique ID was written on the cassette before beginning the test on each eligible child. If the child was right-handed, the 4th finger on the left hand was chosen for finger pricking. Likewise, the right 4th finger was chosen if the child was left-handed. The result was read within 20 min and recorded on the case record form.

### Diagnosis of malaria by microscopy

In both settings, a research blood slide was taken from each participant at the time of testing for expert microscopy. Thick blood films were prepared on each glass slide using 10 µL of blood, evenly spread to cover an area of 15 × 15 mm of the slide. The smear was stained with 1% Giemsa for 25 to 30 min and then examined under oil immersion with a light microscope (magnification 100×). The slides were double-read by trained microscopists. Parasite species were estimated by counting the number of parasites per 200 white blood cells (WBCs) in the thick film. Counts of gametocyte were taken against 500 WBCs in determining gametocyte density per microlitre blood [18].

A sample was considered negative only after 200 high power fields had been read. Parasite counts were converted to parasites per µL, with the assumption that there was an average of 8000 leukocytes per µL of blood. In cases where there was more than 50% discrepancy between parasite counts from the two microscopists readings or when there was a discrepancy qualitatively (positive or negative), a third microscopist read the slide and his/her reading was final and used in the analysis of parasite density. As part of quality control monitoring, randomly selected stained slides from each batch of slides were given to an independent expert microscopist to read. The slides were read on the same day after a day’s field work. Two nurses from hospital were attached to the project. When children were found to have malaria parasitaemia after reading the research slides, they were notified for follow-up purposes.

### Statistical analysis

Data from participants were recorded on specified data collection tools, and these were verified for consistency, completeness and accuracy at the study site. All data collected were entered into EpiData 3.1 software, cleaned and exported to STATA version 14.1 for analysis. Simple frequency and percentages were used for categorical variables. Statistical significance was considered based on a precision of 5% and a confidence interval of 95%. The results were displayed in tables and graphs. IDs were assigned to participants for data entry into the database. This was to ensure confidentiality since their names were not captured into the database. The PI was the only one who had access to the entered database, which was encrypted with a special password. This was to ensure that data are secured and properly managed.

## Results

### Description of the study population

Eight-hundred and seventy-eight (878) children aged 6 to 59 months with complaint of fever or history of fever were recruited for the study with 439 children each from the intervention and comparator arm. The overall mean age of the children was 3.0 ± 1.4 months. Of the 878 children, 495 (56.4%) were males. More than half of the children 680 (77.5%) had normal body temperature (< 37.5 °C) at the time of reporting to health facilities. Most of the caregivers 763 (86.9%) owned a LLIN and 526 (68.9%) of children had slept inside an LLIN the night before the survey.

The overall mean age of the mothers/caregivers was 31.6 ± 8.9 years. There were more mothers/caregivers with junior high school education in the intervention arm than the comparator arm (47.6 vs. 40.3%). More than half of the mothers/caregivers (65.4%) were married in both study arms. Most mothers/caregivers (81.4%) were Christians. More than half of the mothers/caregivers (57.3%) were of Ewe ethnic extraction and 43.5% were traders.

The overall mean age for clinicians was 28.9 ± 5.5 years. More than half of the clinicians (66.7%) were under 30 years of age. Most of the clinicians (91.7%) were males. All clinicians (100%) were first-degree holders and majority (83.3%) were Physician Assistants. Most (83.3%) were single and more than half (66.7%) had less than 2 years of work experience.

### Prevalence of malaria infection by microscopy and RDT

**Fig. 1:**
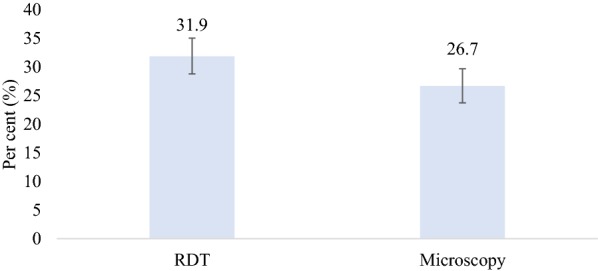
Prevalence of malaria by RDT and microscopy

Of the 878 children recruited, 280 (31.9%) and 234 (26.7%) tested positive for malaria RDT and expert microscopy, respectively. Clinicians in the comparator arm did not send an approximate of 15% of the total eligible children for malaria testing, hence those children were excluded from the study. Overall, the observed prevalence of malaria in the children as indicated by the RDT was significantly higher than that of the microscopy (31.9 vs. 26.7%; p < 0.001) (Fig. 1). The prevalence of malaria by RDT was 31.9% in both study arms but was higher in the comparator arm (28.0%) than the intervention arm (25.3%) based on microscopy (Table 1).

**Table 1:** Prevalence of malaria using RDT and microscopy by study arm

Health facility	Malaria prevalence according to RDT n (%)	Malaria prevalence according to microscopy n (%)
Intervention	140 (31.9)	111 (25.3)
Comparator	140 (31.9)	123 (28.0)
Total	280 (31.9)	234 (26.7)

### Clinicians’ adherence to malaria test results

**Fig. 2:**
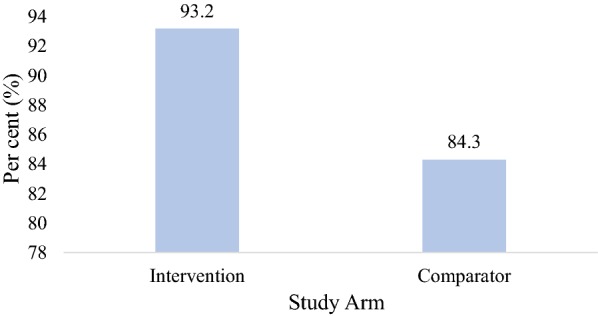
Clinicians adherence to malaria test results

Clinicians’ adherence to malaria test results among children aged 6 to 59 months in the intervention arm was significantly higher than those in the comparator arm (93.2 vs. 84.3%; p < 0.001) (Fig. 2). Out of those who tested positive for malaria, 5.6% were not prescribed with anti-malarial drugs.

### Proportion of children that received diagnoses of malaria following negative test results

In the comparator arm, out of 316 children with negative malaria test results, 67 (21.4%) were still diagnosed with malaria as compared to 25 (8.4%) out of a total of 299 children who tested negative in the intervention arm (Table 2).

**Table 2:** Clinicians’ diagnoses following negative test results

Diagnosis following negative results	Study Arm	
	Intervention n (%)	Comparator n (%)
Malaria	25 (8.4)	67 (21.2)
Other diagnoses	274 (91.6)	249 (78.8)
Total	299 (100.0)	316 (100.0)

### Average time spent at the OPD

**Fig. 3:**
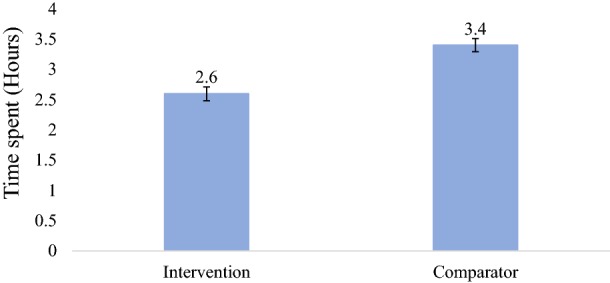
Average time spent (hours) at the health facility

The average waiting time at the OPD of the health facility was significantly lower among those in the intervention arm than in the comparator arm (2.6 vs. 3.4 h; p < 0.001) (Fig. 3).

### Preference of pre- and post-consultation testing and view on waiting time at the OPD

**Fig. 4:**
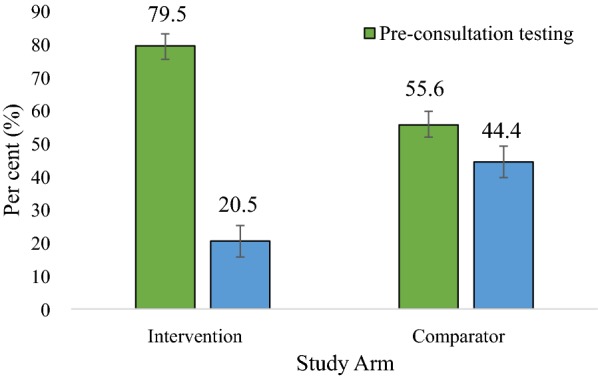
Mothers/caregiver’ preference for pre- and post-consultation

**Fig. 5:**
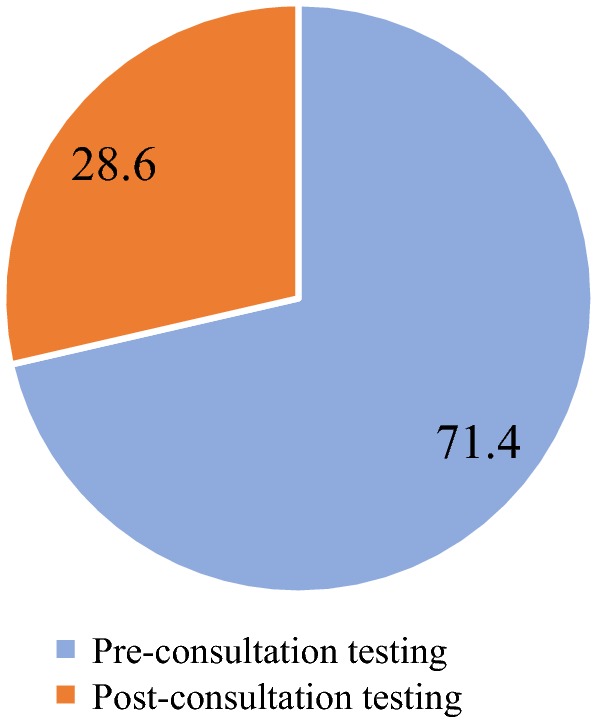
Clinicians’ preference of pre- or post-consultation testing at the intervention arm

**Fig. 6:**
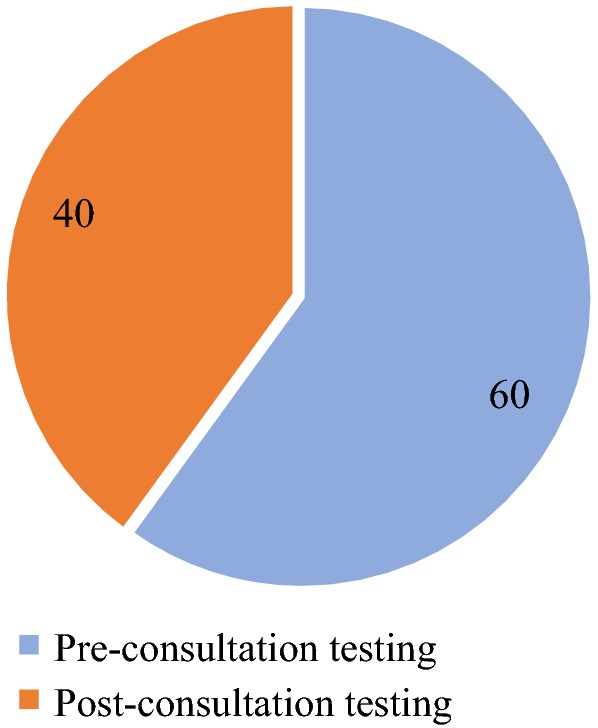
Clinicians’ preference of pre- or post-consultation testing at the comparator arm

**Fig. 7:**
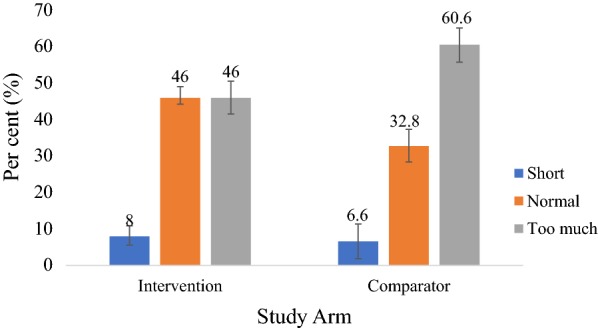
Mothers/caregivers’ view of waiting time at the OPD

A significantly higher proportion of mothers/caregivers with children aged 6 to 59 months preferred to have their children tested before consulting with the clinician (pre-consultation testing) in both study arms (Fig. 4). However, more mothers/caregivers preferred the pre-consultation testing in the intervention arm than in the comparator arm (79.5 vs. 55.6%; p < 0.001). Overall, more than half of the clinicians preferred the pre-consultation testing compared to the post-consultation testing, with a preference of 71.4 and 60.0% in the intervention and comparator arms, respectively (Figs. 5 and 6). A significantly higher proportion of mothers/caregivers viewed the waiting time at the OPD as “too much” in the comparator arm than in the intervention arm (60.6 vs. 46.0%; p < 0.001) (Fig. 7). Even though the difference was not statistically significant, mothers/caregivers who viewed the waiting time as “short” was higher in the intervention arm than in the comparator arm (8.0 vs. 6.6%; p = 0.517) (Fig. 7).

## Discussion

The findings from this study showed that the overall prevalence of malaria among children aged 6 to 59 months with complaints of fever or history of fever based on RDT testing was higher than the prevalence based on microscopy (31.9 vs. 26.7%). The SD BIOLINE Malaria Ag P.F (HRP2/pLDH) test kits were used for the rapid qualitative detection of HRP-II antigen and pLDH from *P. falciparum* in the finger-prick blood sample in the diagnosis of malaria infection. Since the RDT picked up HRP2 antigens, this may have accounted for the increased prevalence observed using RDTs.

The difference in prevalence using different testing methods observed in the current study is similar to that of Lekweiry and others in their study carried out in Nouakchott, Mauritania where a malaria prevalence of 34.9 and 28.9% was found among children aged 6 to 59 months using RDT and microscopy testing, respectively [19].

In Madagascar, Ratsimbasoa et al. [20] also found malaria prevalence at the OPD of 55.8% by RDT testing as compared to 51.6% by microscopy in the same setting. Similarly, a study conducted in four hospitals in the Volta region of Ghana found 56.4% of the children positive for malaria parasites by RDT and 41.7% by microscopy [21]. In the Hohoe Municipality of Ghana, Kweku et al. found the prevalence of malaria as indicated by RDT and microscopy as 28.5 and 16.0%, respectively [22].

The prevalence of malaria as indicated by RDT was higher than microscopy in this current study as was found in the other studies. The difference in malaria prevalence found using the two testing methods (RDT and microscopy) could be due to persistence of circulating HRP2 antigens after malaria treatment, leading to treated patients still being identified as positive cases by RDT [8].

Clinicians’ adherence in the intervention arm was significantly higher than that of those in the comparator arm (93.2 vs. 84.3%; p < 0.001). This finding agrees with that of Asiimwe et al. [23] in Uganda which showed that testing with RDT prior to consultation with the clinician (intervention arm) served as an empowerment tool for clinicians and helped them make diagnosis which they felt was reliable. In Tanzania, Mubi and colleagues revealed that, an increase in adherence which is usually seen in a decrease in anti-malarial prescription in instances where a child tests negative may be an indication of acceptance of RDT as compared to children consulting with a clinician and being asked to go for microscopy testing (comparator arm) [24]. The finding of the current study is consistent with the results obtained by Bilal et al. [20] in Sudan. In their study, they reported 98.4% adherence to test results. The reason for the increased clinicians’ adherence in the intervention arm could be a confirmation that clinicians have more confidence in RDT results and were therefore more likely to adhere to the test results as compared to the routine standard practice of microscopic testing.

The current study found that in the intervention arm, 8.4% of the children aged 6 to 59 months reporting with fever or a history of fever were still diagnosed with malaria following a negative RDT result, compared to 21.2% in the comparator arm following microscopy test results. The findings differ from what was found by Manyando et al. [21] in Zambia. In that study, it was reported that, two out of three (68.6%) of the children who tested negative with RDT were diagnosed with malaria. Similarly, in Malawi, 58% of children with negative RDT results were diagnosed with malaria [22]. In Ghana, Ansah et al. [8] reported that 46.0% of the patients with negative RDT results were still treated for malaria.

The reason for the lower proportion of children under 5 years diagnosed with malaria following a negative RDT in the intervention arm in this current study compared to earlier studies could be that over time, clinicians may have begun to have more confidence in RDT results than they used to have since the other studies were carried out much earlier. However, the reason why clinicians still diagnosed children with malaria following negative RDT results could be partly attributed to lack of capacity by clinicians to adequately investigate other causes of fever [18, 25].

With regard to the average time spent at the OPD, mothers/caregivers’ children in the intervention arm spent less time compared to those reporting at the comparator arm (2.6 vs. 3.4 h). The time spent by patients reporting to the intervention health facility is similar to that spent by patients in an earlier study conducted by Ogunfowokan and Mora [25] in Nigeria. In the Nigerian study, the average time spent was 2.7 h when the children were tested before consulting with the clinicians [25]. The findings of this present study are also similar to those obtained in the study conducted by Naidoo and Mahomed in KwaZulu-Natal. In that study, it was observed that even though the time difference was not much, their intervention significantly reduced the waiting time at the health facility (from 11.9 to 10.0 min; p = 0.03) [23]. Presenting test results to the clinician in the intervention arm made it easier for them to arrive at diagnoses and treat and this possibly explains why the time spent in the intervention arm is shorter as compared to the comparator study arm where the child sees the clinician who then tries to rule out malaria by requesting for a test [23].

In this study, the proportion of mothers/caregivers who viewed the waiting time at the OPD as “too much” was significantly lower in the intervention arm than in the comparator arm (46.0 vs. 60.6%; p < 0.001). The current study confirms the dissatisfaction of mothers/caregivers of children aged 6 to 59 months with the long waiting time in the health facility as a result of seeing the clinician before requesting for the test, as practiced in the comparator arm [14]. In a similar study at the OPD of University of Port Harcourt Teaching Hospital in Nigeria, the association between the level of satisfaction and overall time spent at the health facility was statistically significant (p > 0.001) [24]. In this current study, even though the difference was not statistically significant, the proportion of mothers/caregivers who viewed the waiting time as “short” was higher in the intervention arm than in the comparator arm (8 vs. 6.6%; p = 0.517), and this seems to indicate that pre-consultation testing positively influences how patients view their time spent at the health facility. This agrees with the study findings of at National Hospital Abuja, Nigeria which revealed that good impression about patient–clinic encounter time (normal or short visit time) was significantly associated with satisfaction (Fisher’s exact Chi-square = 28; *p *< 0.001) [25].

### Strength and limitations

Clinicians were interviewed after the estimated sample size of the mothers/caregivers were recruited, to avoid them being aware that the test results the children presented during the consultation were to determine their level of adherence to the test results. This was done to avoid bias.

The medical superintendents in charge of the hospitals, were however made aware of this when permission was being sought and were assured of anonymity and confidentially of the study. It may however, be likely, though remotely so, that they might have informed some clinicians of the aim of the study.

Additionally, baseline data on patients waiting time were not collected in both hospitals prior to the introduction of pre-consultation testing in the intervention arm. Even though the hospitals were paired according to certain characteristics such as size, case load, staffing, and availability of testing facilities, there was a possibility of other differences in the two hospitals which might have contributed to the longer consultation time in the comparator arm.

## Conclusion

Pre-consultation testing for malaria at an OPD significantly improved clinicians’ adherence to malaria test results, shortened patient waiting time and led to an overall patient satisfaction compared to post-consultation testing in the Northern Volta region of Ghana. Pre-consultation testing resulted in a significant reduction in clinicians’ diagnoses of malaria following negative test results. Clinicians and caregivers of children aged 6 to 59 months in both settings indicated a preference to have children tested at OPD of the hospitals before consulting with a clinician. Inclusion of RDT for children aged 6 to 59 months with fever at the triaging area of OPDs will lead to an improvement in the quality of diagnosis and treatment of malaria.

## Data Availability

The data and detailed protocol can be made available upon request from the corresponding author.

## Abbreviations

ACT: Artemisinin-based combination therapy
A–L: Artemether–lumefantrine
CDC: Centers for Disease Control and Prevention
CHPS: Community-Based Health Planning and Services
DNA: Deoxyribonucleic acid
DPQ: Dihydroartemisinin–piperaquine
GCP: Good Clinical Practice
GHS: Ghana Health Service
GMIS: Ghana Malaria Indicator Service
GSS: Ghana Statistical Service
Hb: Haemoglobin
HBB: Human-Beta-Globin
HRP: Plasmodium histidine-rich protein
ICS: Immunochromatographic strip
ICH: International Conference on Harmonisation
LDH: Lactate dehydrogenase
LLIN: Long-lasting insecticidal net
MOH: Ministry of Health
RDT: Malaria Rapid Diagnostic Test
NMCP: National Malaria Control Programme
OPD: Out-Patient Department
PCR: Polymerase chain reaction
RDT: Rapid Diagnostic Test
REC: Research Ethical Committee
TAT: Turn-around time
T3: Test, treat and track
UNICEF: United Nations International Children’s Emergency Fund
VRHD: Volta Regional Health Directorate
WBC: White blood cells
WHO: World Health Organization
